# Myeloproliferative neoplasms – blurring the lines between cancer and chronic inflammatory disorder

**DOI:** 10.3389/fonc.2023.1208089

**Published:** 2023-06-09

**Authors:** Eli M. Soyfer, Angela G. Fleischman

**Affiliations:** ^1^School of Medicine, University of California, Irvine, Irvine, CA, United States; ^2^Division of Hematology/Oncology, University of California (UC) Irvine Health, Irvine, CA, United States; ^3^Chao Family Comprehensive Cancer Center, University of California, Irvine, Irvine, CA, United States

**Keywords:** inflammation, myeloproliferative neoplasm, autoimmunity, cytokines, clonal hematopoiesis

## Abstract

Myeloproliferative Neoplasm (MPN) is a group of chronic blood cancers that arise from a hematopoietic stem cell (HSC) clone with somatic mutations causing constitutive activation of myeloid cytokine receptor signaling. In addition to elevated blood cell counts, MPN typically presents with increased inflammatory signaling and inflammation symptoms. Therefore, while being a clonally derived neoplasm, MPN has much in common with chronic non-cancerous inflammatory conditions, such as rheumatoid arthritis, lupus, and many more. MPN and chronic inflammatory disease (CID) share similar chronicity, symptoms, dependency on the immune system, environmental triggers, and treatments. Overall, we will highlight the similarities between an MPN and CID. We highlight that while MPN is classified as a cancer, its behavior is more aligned to that of a chronic inflammatory disease. We propose that MPN should inhabit a fluid/spectrum between auto-inflammatory disease and cancer.

## Graphical Abstract

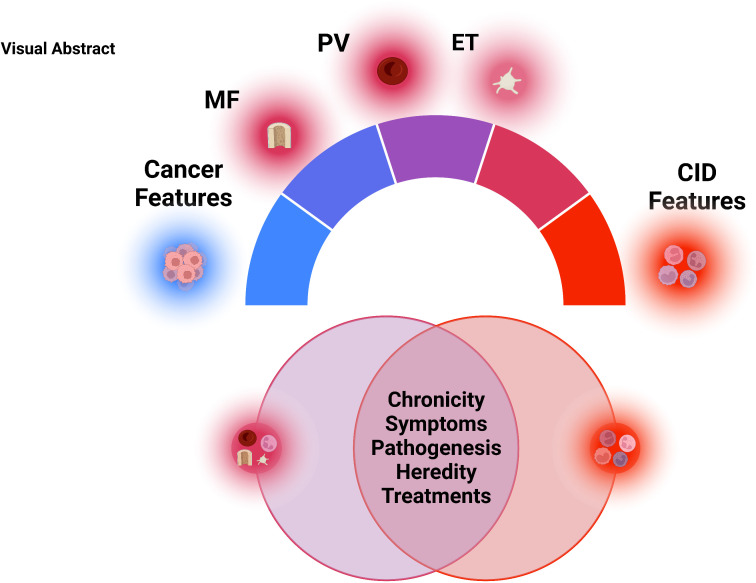

## Introduction

Classical/Philadelphia chromosome negative myeloproliferative neoplasms (MPN) are a group of hematologic malignancies including polycythemia vera (PV), essential thrombocythemia (ET), and primary myelofibrosis (PMF). Each subset (PV, ET, PMF) has its unique clinical features with a unifying theme of somatic acquisition of a mutation in either Janus Activated Kinase 2 (*JAK2^V617F^*) (1–5), Calreticulin (*CALR*) (6, 7) or Thrombopoietin Receptor (*TPOR*, *MPL*) in a Hematopoietic Stem Cell (HSC). *JAK2^V617F^* mutations can be seen in all three subtypes of MPN, whereas *CALR* or *MPL* mutations are restricted to ET and PMF. This change leads to an overproduction of any combination of white cells, red cells, and platelets, with major clinical consequences including increased risk of thrombosis, and constitutional symptoms. Development of MF, primary (PMF) or secondary to PV/ET, results in cytopenias and risk of transformation to acute leukemia. Chronic inflammation is a hallmark feature of MPN, most notably PMF, which plays an integral role in multiple aspects of its pathobiology, including symptomatology, thrombosis, disease progression, and heightened cardiovascular disease risk (8–11). Although MPN is currently classified as a malignancy, many of the aspects of the disease are more like a chronic inflammatory condition rather than a cancer.

**Figure 1:**
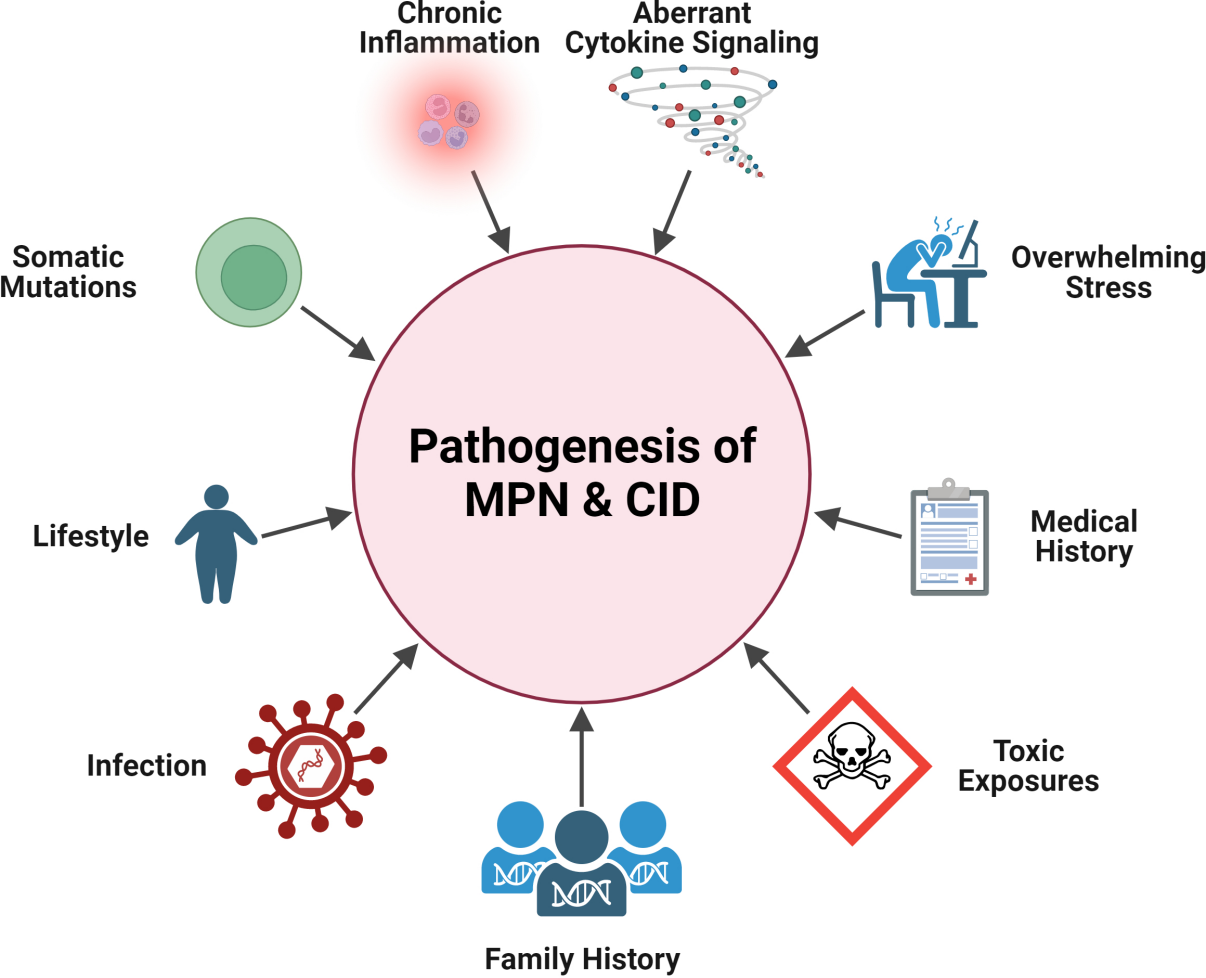
Shared pathogenesis features in MPN and CID. Pathogenic elements that are in common between MPN and CID: aberrant cytokine signaling, chronic inflammation, somatic mutations, general stress, lifestyle (e.g., obesity), medical history (e.g., MPN patient having pre-existing CID), family history, and exposures (infectious, toxic). Created with BioRender.com.

In the sections below, we will highlight specific clinical and pathobiological overlaps between inflammatory disease and each subset of MPN, summarized in **Figure 1**.

### Chronicity

Inflammatory disease is the result of immune-mediated damage of self-tissues. It is characterized by the presence of chronic inflammation mediated by loss of self-tolerance mechanisms (commonly from genetic mutations) that would normally regulate self-reactive lymphocytes, or inappropriate activation of innate immune cells commonly triggered by certain environmental exposures (e.g., specific foods, chemicals). Inflammatory disease severity increases with chronic exposure to inflammatory stimuli, causing tissue damage and fibrosis.

MPNs are also chronic in nature. The MPN driver clone emerges decades prior to diagnosis (12, 13), and in some cases arises *in utero* (14). Normal life expectancy is possible in essential thrombocytosis (ET) and polycythemia vera (PV) (15, 16), but in general patients with these diseases have a shorter life expectancy than their age matched counterparts (17). Much of the morbidity and mortality in MPN stems from cardiovascular pathology which is characterized as an inflammatory disease (18). MPN patients are at increased risk for both arterial and venous thromboses. A large population based study found the Hazard Ratios (HRs) of arterial thrombosis among MPN patients compared to controls at 3 months, 1- and 5 years post diagnosis to be 3.0, 2.0, and 1.5, respectively (19). The corresponding HRs for venous thrombosis were 9.7, 4.7, and 3.2, and for myocardial infarction the HRs were 2.5, 1.8, and 1.4 (19).

A large population study found that presence of at least one cardiovascular disease (CVD) risk factor predicted higher risk for thrombosis among MPN patients (20). Accordingly, reduction of CVD risk factors is paramount in MPN, and as such is listed as the initial item on the intervention list for MPN’s in the National Comprehensive Cancer Center Network (NCCN) guidelines. However, there are no established strategies for how this risk reduction should be achieved specifically in MPN patients.

Primary Myelofibrosis (PMF), although also a chronic disease, has significantly worse prognosis than ET or PV. Even within PMF there is a wide range of life expectancy and because of this there are multiple scoring tools available to help clinicians prognosticate in PMF. Although the thrombotic risk and CVD risk is present in PMF, goal of care in PMF are more focused on improving symptom burden, splenomegaly and cytopenia as well as surveillance for transformation to acute myeloid leukemia.

### Symptoms

#### Symptom overlap between MPN and CID

**Figure 2:**
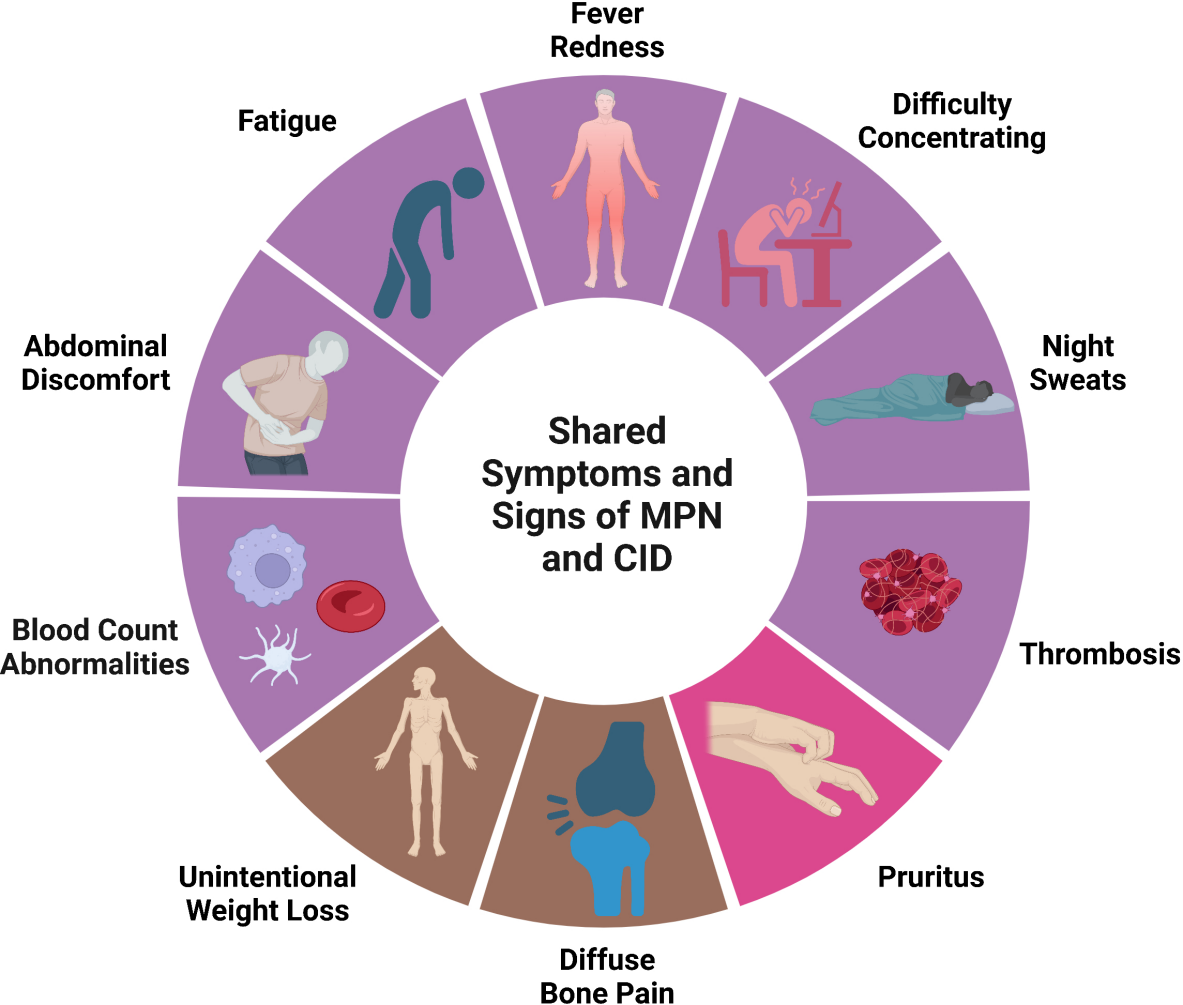
Shared signs and symptoms in MPN and CID. Symptoms and signs that can be seen in both MPN and CID. Purple represents signs/symptoms seen in all MPN subtypes, bright pin represents signs/symptoms seen in PV primarily, brown represents signs/symptoms seen primarily in PMF. Created with BioRender.com.

There is significant overlap between signs and symptoms in MPN and auto-inflammatory disease (**Figure 2**). Symptoms are clearly the most impactful clinical feature of PMF with extreme fatigue being prevalent. Weight loss, night sweats, and fever can also be seen in PMF along with abdominal pain from splenomegaly. Symptoms are not restricted to patients with PMF. Headaches, numbness/tingling, and pruritis can also be debilitating in PV and ET. The overall symptom burden is a significant morbidity associated with MPN which negatively affects quality of life (21), leads to impaired work productivity (22, 23), and the need for medical disability leave (24). The central importance of symptom burden in MPN is reflected in the widespread use of the MPN Symptom Assessment Form (MPN-SAF), an internationally validated objective scoring tool to quantify symptom burden in MPN patients (25, 26). Reduction in MPN-SAF score is a key endpoint in MPN clinical trials and reduction in symptom burden was the outcome that led to the FDA approval of the JAK inhibitors ruxolitinib (27), fedratinib (28), and pacritinib (29) for myelofibrosis.

The prevalence and severity of symptoms differ by MPN subtype and even within each MPN subtype symptoms can be variable. For example, a prospective evaluation of 1470 MPN patients, including 622 ET, 519 PV, and 329 PMF patients identified five symptom clusters in PV and ET, respectively, and four clusters in PMF (30). Clinical variables including age, language, gender, the presence of laboratory abnormalities, spleen size, history of hemorrhage, and MPN-SAF score. Despite symptom management being regarded as a high therapeutic priority, symptoms are still inadequately addressed particularly for patients with ET and PV (31).

Symptoms are also integrated into disease scoring tools in autoimmune disease, including the Systemic Lupus Erythematosus Disease Activity Index (SLEDAI) (32) in SLE, the Simplified Disease Activity Index (SDAI) and Clinical Disease Activity Index (CDAI) (33, 34) in Rheumatoid Arthritis, and the Sjögren’s Tool for Assessing Response (STAR) (35) in Sjögren’s syndrome.

#### Symptom management strategies

The effectiveness of currently available therapies may be in part due to their ability to reduce the inflammatory state of MPN patients. Ruxolitinib, a JAK1/2 inhibitor Food and Drug Administration (FDA) approved for MF patients and for PV patients intolerant or resistant to hydroxyurea, has anti-inflammatory properties and its administration has been correlated with reductions in plasma levels of C reactive Protein (CRP), interleukin-1 receptor-alpha (IL-1Rα), Macrophage Inflammatory Protein-1-beta (MIP-1β), Tumor Necrosis Factor-alpha (TNF-α) and interleukin-6 (IL-6) (8). However, plasma cytokines remain abnormal following ruxolitinib (36), indicating that JAK inhibitors are not sufficient to normalize cytokines (36). Moreover, JAK inhibitors are not without significant side effects including thrombocytopenia, anemia, increased risk of skin cancer, and immunosuppression. There remains an unmet need in MPN care for low-risk treatment options that improve or control disease related inflammation.

#### Symptom burden and obesity

Obesity causes chronic low-grade inflammation (37), body mass index (BMI) is a modifiable variable. A U-shaped association between BMI and Total Symptom Burden was observed in a combined analysis of two large cross-sectional surveys, the Danish Population-based Study, MPN health survey (n = 2044), and the international Fatigue Study (n = 1070), with significantly higher mean symptom scores for underweight and obese patients relative to normal weight (38). Interestingly, in an Israeli population study obesity (BMI ≥ 95th percentile) in adolescence significantly predicted increased risk of PMF with HR of 1.81 (95% confidence interval 1.13-2.92, P = 0.014) (39).

In PV patients, the PV-NET Real World Study was conducted to evaluate the impact of Charlson Comorbidity Index (CCI) and BMI on treatment success and survival in 530 PV patients. The study concluded that CCI/BMI can influence the choice between therapies (interferon vs JAK inhibitor), and that quantification of body composition and control of comorbidities can improve PV outcome (40, 41). Therefore, a focus on maintaining a healthy BMI should be emphasized in all subsets of MPN.

#### Inflammation, the driving symptom of MPN and CID

Inflammation appears as pain (joint or diffuse bone), redness, fatigue, urticaria, fever, and stiffness. These symptoms and any disease-specific signs (e.g., malar rash as seen in lupus) would prompt a blood workup, genetic screening, and imaging for indicators of inflammatory disease. The etiology of inflammation in MPN is likely multifactorial. The *JAK2^V617F^* mutant cells not only produce excessive inflammatory cytokines themselves, but also induce other bystander cells to produce inflammatory cytokines (42).

Chronic inflammation is responsible for much of the symptom burden in MPN (43), and also contributes to disease development and progression by promoting expansion of the neoplastic clone (44, 45). Importantly for symptom management, specific symptoms have been correlated with elevated levels of specific inflammatory biomarkers (43). In treatment-naive PMF patients, increased levels of Interleukin-8 (IL-8), IL-2R, IL-12, IL-15, and Interferon gamma-induced protein 10 (IP-10) were independently predictive of inferior survival (10).

Each subset of MPN may have its own distinct inflammatory signature. For example, ET has a specific inflammatory cytokine signature consisting of Eotaxin, GRO-α, and EGF (46). GRO-α and EGF in ET patients were associated with disease transformation in initial sample collection (GRO-α) or longitudinal sampling (EGF). Therefore, addition of cytokine profiling could potentially add prognostic value for predicting transformation from ET to PMF.

## Clonality in MPN and CID

The identification of somatic mutations leading to expansion of a clone transitioned the nomenclature of ET, PV, and PMF from a *disorder* to a *neoplasm*. However, presence of a clone with an MPN driver mutation can be seen in humans without a clinical MPN. *JAK2^V617F^* is the 5^th^ most common mutation seen in clonal hematopoiesis of indeterminate potential (CHIP) (47). Screening of almost 20,000 Danish citizens revealed presence of somatic *JAK2^V617F^* and *CALR* mutations in 3.2 and 0.16 percent of the population (48). Screening of >250,000 people who have submitted samples to 23andme personal genome service who denied a history of MPN revealed *JAK2^V617F^* in approximately 0.2% of these samples (49).

The clonal architecture of MPN can be complex, with additional mutations acquired in the MPN clone either before or after the MPN driver mutation, or in separate clones from the MPN driver clone (50, 51). Mutations in high risk genes including *ASXL1, EZH2, SRSF2* and *IDH* identifies PMF patients who are at risk for premature death or leukemic transformation (52). Patients with an *ASXL1* mutation on its own had no increased prognostic to a worsening outcoming, unless when compounded with one of the high-risk genes (53).

A perceived distinguishing factor between hematologic malignancy and inflammatory disease is somatic mutations, present in hematologic malignancy but absent in autoimmune/inflammatory disorders. However, this line is also blurring. Somatic mutations in hematopoietic cells are being identified in inflammatory conditions. A prime example of somatic mutations of myeloid lineage cells causing a severe inflammatory disorder is VEXAS (vacuoles, E1 enzyme, X-linked, autoinflammatory, somatic) syndrome (54). VEXAS is caused by somatic mutations in UBA1 that result in systemic inflammation and progressive bone marrow failure that presents late in life.

Patients with other non-malignant autoimmune disorders harbor somatic mutations in T cells. Individuals with newly diagnosed, untreated Rheumatoid Arthritis (RA) are found to harbor somatic mutations in clonally expanded CD8+ T cells (55). Somatic mutations in the CD8+ T cell compartment have also been identified in patients with myasthenia gravis (56).

## Overlapping populations of MPN and autoimmune disease

There is a significant overlap in the autoimmune disease and MPN patient populations. A prior history of any autoimmune disease was found to be associated with a significantly increased risk of MPN with an Odd’s Ratio (OR) of 1.2 (57). Another study found a significantly increased risk of MPNs in subjects with a prior history of any autoimmune disease (OR=1.86) (58). MPN is likely underappreciated in patients with autoimmune disease, as elevated platelets could be attributed to reactive thrombocytosis in this patient population. Analysis of patients with thrombocytosis (platelets > 450) in an inflammatory bowel clinic revealed that 23% of them harbored JAK2^V617F^ mutations (59).

It is important to distinguish the myeloproliferative neoplasm PMF from autoimmune MF (AIMF). While both conditions cause fibrosis, cytopenia, and elevated inflammatory cytokines, AIMF will present with serological evidence of autoantibodies and lack of MPN driver mutations (60). However, PMF patients may contain autoantibodies, especially from a prior autoimmune disease, requiring a physician to not rely on this test alone (61).

## Familial predisposition in CID and MPN

For various auto-immune conditions, a high degree of heritability is seen among family members of the affected patient, especially in first-degree relatives (62, 63). This can be due to several conditions centered around similar genetic makeup and environmental exposures. There is also a familial predisposition to acquire MPN. A study involving 35,037 hematologic malignancies patients and 93,199 of their first degree relatives found an increased risk of developing AML (RR 1.53), MDS (RR 6.87), and the MPN subtypes PV (RR 7.66) and ET (RR 6.3) if a family member has the disease (64). A search for the genetic basis of the predisposition to acquire MPN has revealed single nucleotide polymorphisms (SNPs) associated with MPN, for example the germline haplotype (GGCC, referred to as “46/1”) encompassing the 3’ region of the JAK2 gene is associated with a three- to four-fold risk of MPN (65). Interestingly, this same JAK2 46/1 haplotype also increases susceptibility to Crohn’s disease, an autoimmune inflammatory bowel disease (66). However, the genetic predisposition to acquire MPN has yet to be fully elucidated.

## Infectious triggers for CID and MPN activation

A common activator of the disease state in MPN and autoimmune disease may be exposure to a severe inflammatory stimulus such as infection. An emerging theme among mutations associated with myeloid malignancies and clonal hematopoiesis of indeterminate potential is resistance to chronic inflammation. For example, many chronic inflammatory stimuli, including Interferon-gamma (IFN-γ (67) and TNF-alpha (TNFα) (45, 68) augment the selective advantage of clones with myeloid malignancy associated mutations in mouse models. However, the impact of an acute inflammatory stressors on the emergence of neoplastic clones is an area less studied. An acute inflammatory stimulus may allow for the expansion of a neoplastic clone to reach a critical threshold after which chronic low grade inflammation, either from environmental sources or driven by the neoplastic clone itself maintains the selective pressure in favor of continued expansion of the clone. Further research, both epidemiologic and mechanistic is required to elucidate the impact of acute infectious stressors on the development of MPN.

Epidemiologic data support the notion that infection may promote development of MPN. A large Swedish population-based study demonstrated that a history of infection was associated with an increased risk of AML and MDS (69). Using the Surveillance, Epidemiology and End Result (SEER)-Medicare database Titmarsh et al. found that MPN was significantly associated with a history of cellulitis (70). Infection is a well-established trigger for autoimmune disease. There are different mechanisms by which an infection may trigger autoimmunity, including molecular mimicry, epitope spreading, and bystander activation. Exposures to specific pathogens have been associated with specific autoimmune disease. Increased amounts of antibodies formed against various bacteria have been linked to driving lupus (71). Epstein-Barr virus infection have been implicated in the pathogenesis of multiple sclerosis (72).

## Treatment overlaps in MPN and CID

The most notable therapy overlap is the use of JAK inhibitors as a mainstay of treatment in both MPN and autoimmune disease. While in MPN, JAK inhibitors suppress constitutively active JAK/STAT signaling, their ability to reduce inflammation in general may be their broader mechanism of action. The JAK inhibitor ruxolitinib reduces inflammation, however not down to the level of normal (36). This suggests that additional anti-inflammatory drugs, such as those that target the NF-ĸB pathway could work synergistically with JAK inhibitors to quell inflammation in MPN. Prior to the JAK inhibitor era, anti-TNF agents had been investigated in MPN (73), however not nearly to the depth as anti-TNF agents in autoimmune disease. This highlights the common goal in MPN and CID therapy is to reduce inflammation. Further treatment that addresses anti-inflammation specifically (in combination with JAK inhibitors) is being studied, specifically the targeting of TNF-α receptors 1/2 (TNFR1/2) to help control constitutive symptoms (74).

Interferon-alpha (IFNα) is used in MPN in multiple formulations and has the potential of inducing a molecular response (75–77). IFNα can exacerbate autoimmune conditions (78), demonstrating that some drugs used for MPN may be contraindicated in autoimmune disease. However, other interferons are used for autoimmune disease. Evidence supports an anti-inflammatory and beneficial role of IFNβ locally in the joints of patients with rheumatoid arthritis and in murine arthritis models, and many patients with multiple sclerosis show a clinical response to recombinant IFNβ (79).

## Lifestyle modification in MPN

Interventions such as physical activity and yoga have been implicated in reduction of symptom burden and improvement in quality of life in MPN (80–82). A role between increased inflammation and development of depression has been characterized in recent years, encouraging the development of depression reduction techniques (83, 84). Pilot studies with the mindfulness meditation app *Calm* suggest that this may be effective in reducing depression and anxiety symptoms in MPN patients (85–87). A randomized online yoga intervention demonstrated small effects on sleep, pain, and anxiety as well as a moderate effect on depression (80). Ultimately, a multimodal lifestyle approach (diet, exercise, mindfulness) is likely the optimal intervention in MPN, but first we must rigorously interrogate the impact of each modality on its own before combining them into a holistic lifestyle approach to improving health in MPN.

Dietary management may represent a low-risk way to optimize cardiovascular health and reduce inflammation in MPN. The Mediterranean diet, characterized by increased consumption of extra virgin olive oil (EVOO), nuts, legumes, vegetables, fruits, fish, and whole grain products, has proven beneficial in modifying subclinical inflammation in diseases where chronic subclinical inflammation plays a key role (88). Specifically, the randomized interventional PREDIMED (Prevención con Dieta Mediterránea) study demonstrated that a Mediterranean diet supplemented with EVOO reduced the incidence of major cardiovascular events (89). Numerous longitudinal cohort studies show that a Mediterranean style eating pattern was associated with lower risk for cardiovascular disease (CVD), explained in part by reduction in CVD risk factors, most notably inflammation (90). We performed two pilot interventional studies to establish the feasibility of a Mediterranean diet in MPN (Mendez Luque et al, manuscript in preparation). We found that MPN patients can alter their diet toward a Mediterranean diet eating pattern (91). We did not observe significant changes in plasma inflammatory cytokines or gut microbiome over the 10-week intervention (92), although analysis was limited due to the small sample size. Upcoming studies with a larger cohort of MPN patients are required to rigorously test the impact of a Mediterranean diet on inflammatory biomarkers and symptom burden.

## Conclusions

Approaching MPN as a disease that lies at the intersection of a neoplasm and chronic inflammatory disorder may better serve patients and the physicians who treat them. Addressing inflammation in MPN will likely reduce symptom burden, ameliorate some of the inflammation-driven morbidity associated with the disease, and potentially blunt disease progression. It is important, however, to note that other treatment modalities beyond reduction of inflammation are necessary to eradicate the neoplastic clone. Investigating MPN through the scientific lens of an inflammatory disease in addition to a cancer may reveal important aspects of MPN pathogenesis that may be missed by viewing MPN through cancer blinders.

## Author contributions

ES and AF together wrote the article and edited it. ES created figures. All authors contributed to the article and approved the submitted version.
